# Dudleigh Oscar (John) Topp FRCPsych, MBBS, MFCM, DPM: Formerly Principal Medical Officer, HM Prison, Brixton

**DOI:** 10.1192/pb.bp.116.054502

**Published:** 2017-02

**Authors:** John Topp

**Figure:**
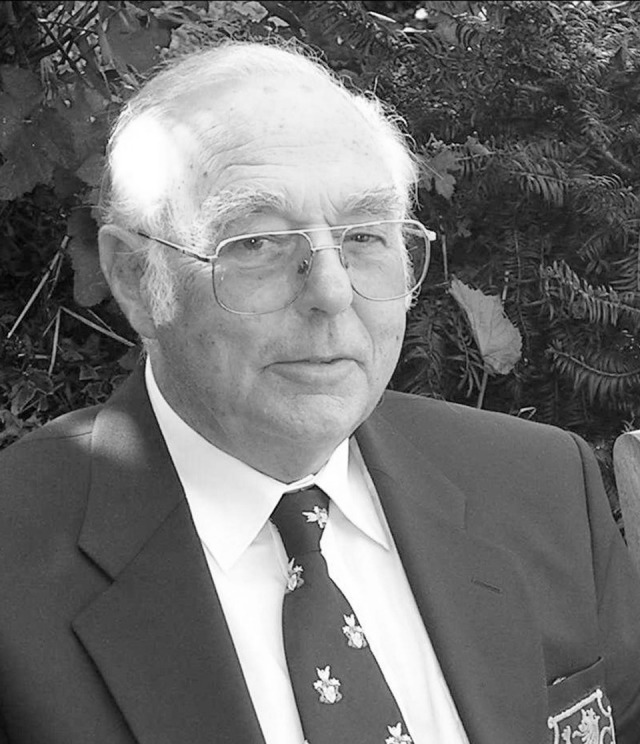


John Topp, who died on 23 February 2016, was a Principal Medical Officer who was one of the first to draw attention to the increased rate of suicide among the prison population. In the late 1970s, he showed that suicide was three times as common in prisoners as in the general population, that it was most common in those with sentences longer than 18 months and that the greatest risk was in the first few weeks in custody.^1^ He subsequently advised on suicide prevention in prisons.

Topp always felt deeply that the prison medical services were often unfairly maligned. He knew that the antiquated estate and restrictive budget did nothing to enhance the practice of modern medicine; yet, he felt that on the whole prisoners were given good care within these constraints. He found the hospital officers of the Prison Medical Service to be generally excellent but felt that many were denied opportunities to achieve the nursing qualifications they desired. Much criticism, he felt, would have been spared if training had been far better resourced and achievement suitably rewarded. Nevertheless, his view was that although the service was not superficially attractive, for those prepared to put effort and interest into it, there could be considerable work satisfaction – something he enjoyed himself despite much frustration.

Dudleigh Oscar Topp, always known as John, was born on 11 September 1924 in Hove, the son of a bank manager. A practising Roman Catholic, he was educated at the Xaverian College in Brighton and attended King's College London, from where he went to Charing Cross Hospital Medical School, qualifying in 1949. After house posts at Oldchurch Hospital, Romford, he served as a captain in the Royal Army Medical Corps in Northern Ireland. He joined the Prison Medical Service in 1953 at Wakefield prison and later became Senior Medical Officer at Brixton prison, from where he was promoted to Principal Medical Officer to serve regionally and centrally, administrating various aspects of the prison medical services.

He retired on medical advice in 1984 but remained actively interested in prison affairs and wrote books on the history of the Prison Medical Service. His particular regret was that the Prison Medical Association, which he founded towards the end of his career with the object of promoting the highest standards of medical care in English prisons, did not survive his retirement. However, he lived to learn of the inception of an Academy of Prison Medicine which naturally had his full approval.

Living latterly in Weymouth, where he had the close companionship of two local retired hospital chief officers, he became the founder president of the Pickering Society, a nationwide group of retired hospital prison staff of all categories. It was named after a respected director of prison medical services under whom most of them had served.

Soon after qualifying, John married his childhood sweetheart Joyce Stoner, a nurse from Brighton. They had four daughters, three of whom trained as nurses at the Westminster Hospital, where two of them met and married doctors. There are now eight grandchildren and five great-grandchildren. Tragically, Joyce died in 1975 of carcinoma of the stomach, following which he married her friend Peggy Lange, then a Principal Nursing Officer in Sunderland.
